# Long-term non-progression in children with HIV: estimates from international cohort data

**DOI:** 10.1097/QAD.0000000000004136

**Published:** 2025-02-04

**Authors:** The European Pregnancy and Paediatric Infections Cohort Collaboration (EPPICC):, Charlotte Jackson, Siobhan Crichton, Ali Judd, Alasdair Bamford, Philip Goulder, Nigel Klein, Laura Marques, Paolo Paioni, Andrew Riordan, Vana Spoulou, Vinicius Adriano Vieira, Santa Ansone, Elena Chiappini, Sophie Le Coeur, Luminita Ene, Luisa Galli, Carlo Giaquinto, Tessa Goetghebuer, Claudia Fortuny, Suparat Kanjanavanit, Magda Marczynska, Marisa Navarro, Lars Naver, Nicole Ngo-Giang-Huong, Yulia K. Plotnikova, Aleksey A. Plynskey, Jose Tomas Ramos, Irina Raus, Vladimir Y. Rozenberg, Anna V. Samarina, Elisabeth H. Schölvinck, Natalia Vasylenko, Alla Volokha, Intira Jeannie Collins, Ruth Goodall

**Affiliations:** a MRC Clinical Trials Unit at UCL, University College London, UK; b Fondazione Penta ETS, Padua, Italy; c Great Ormond Street Hospital for Children NHS Foundation Trust, London; d Department of Paediatrics, University of Oxford, Oxford, UK; e Centro Materno-infantil do Norte, ULSSA, Porto, Portugal; f Division of Infectious Diseases and Hospital Epidemiology, University Children's Hospital Zurich, Switzerland; g Alder Hey Children's NHS Foundation Trust, Liverpool, UK; h Department of Infectious Diseases, University of Athens, Athens, Greece; i Riga East University Hospital, Latvian Centre of Infectious Diseases, Riga, Latvia; j Italian Register for HIV Infection in Children; k Department of Health Sciences, University of Florence; l Meyer Children's Hospital IRCCS, Florence, Italy; m Institut National d’Etude Demographique (INED), Mortality, Health and Epidemiology Unit, Paris, France; n Institut de Recherche pour le Developpement (IRD), UMI-174/PHPT, Faculty of Associated Medical Sciences, Chiang Mai University, Chiang Mai, Thailand; o ‘Dr Victor Babes’ Hospital for Infectious and Tropical Diseases, Bucharest, Romania; p Division of Pediatric Infectious Diseases, Department of Women's and Children's Health, University of Padua, Padua, Italy; q Hopital Saint-Pierre, Université libre de Bruxelles (ULB), Brussels, Belgium; r Translational Research Network in Pediatric Infectious Diseases (RITIP); s Malalties Infeccioses i Resposta Inflamatòria Sistèmica en Pediatria, Unitat d’Infeccions, Servei de Pediatria, Institut de Recerca Pediàtrica Sant Joan de Déu, Barcelona; t CIBER de Enfermedades Infecciosas (CIBERINFECT), Instituto de Salud Carlos III, Madrid, Spain; u Nakornping Hospital, Chiang Mai, Thailand; v Medical University of Warsaw, Hospital of Infectious Diseases in Warsaw, Warsaw, Poland; w Hospital General Universitario ‘Gregorio Marañón’, Madrid, Spain; x Karolinska University Hospital and Karolinska Institutet, Stockholm, Sweden; y Thailand-France Research Collaboration, Faculty of Associated Medical Sciences, Chiang Mai University, Chiang Mai, Thailand; z Irkutsk AIDS Centre, Irkutsk, Russia; aa Hospital 12 de Octubre; bb Complutense University; cc Instituto de Investigación Sanitaria (i+12); dd CIBERINFEC, ISCIII, Madrid, Spain; ee Kyiv City Center for HIV/AIDS, Kyiv, Ukraine; ff City HIV Centre, St Petersburg, Russia; gg Beatrix Kinderziekenhuis, Hanzeplein 1, Postbus 30 001, 9700 RB Groningen, The Netherlands; hh Perinatal Prevention of AIDS Initiative, Odessa; ii Shupyk National Healthcare University of Ukraine, Kyiv, Ukraine.

**Keywords:** children, Europe, HIV, HIV long-term nonprogression, Thailand

## Abstract

**Objectives:**

To estimate the probability of long-term nonprogression (LTNP) in the absence of antiretroviral treatment (ART) in children with perinatally acquired HIV, and the impact of LTNP definitions on these estimates.

**Design:**

Analysis of longitudinal routine care data (follow-up to 2016) collected through a collaboration of cohorts of children in routine HIV care across Europe and Thailand.

**Methods:**

LTNP was defined as reaching age 8 years without disease progression (defined as an AIDS diagnosis or immunosuppression based on WHO immunosuppression-for-age thresholds, age-adjusted CD4^+^*z*-scores or CD4^+^ counts). ART initiation was treated as a competing risk (children initiating ART before age 8 were not considered to have LTNP). We included children born domestically in six national HIV cohorts (*n* = 2481). Additional analyses included domestic-born children enrolled in national cohorts in infancy (aged <12 months, *n* = 1144, six cohorts), or all domestic-born children in national and nonnational cohorts (*n* = 4542, 18 cohorts). Results were stratified by birth year.

**Results:**

Among children born domestically in national cohorts in 2004–2007, the probability [95% confidence interval (CI)] of LTNP at age 8 years was 10% (6–15%) based on WHO immunosuppression-for-age criteria. This was lower for children born earlier when ART use was less frequent. Results were similar using other immunosuppression thresholds. Estimates were lower when restricted to domestic-born children in national cohorts enrolled in infancy, and higher when including all domestic-born children.

**Conclusion:**

Up to 10% of children born during 2004–2007 had LTNP at age 8. Our findings may help identify participants with LTNP for research into posttreatment control and HIV cure.

## Introduction

In the absence of antiretroviral treatment (ART), a high proportion of children with HIV (CWHIV) who acquired HIV perinatally will experience disease progression to AIDS or death within 2 years [1,2], with risk continuing as they age [3]. However, a minority of children remain clinically well without ART and are termed ‘long-term survivors’ [4,5], ‘slow progressors’, ‘paediatric nonprogressors’ [4–6] or ‘long-term nonprogressors’. Understanding the characteristics of these children may inform estimates of undiagnosed and clinically well children as well as HIV cure research [7] and help to explain why some children maintain high CD4^+^ cell counts during treatment interruptions [8,9]. However, in the current era of early infant diagnosis and universal treatment before disease progression, few studies can explore long-term nonprogression (LTNP) in the absence of treatment. Historical datasets from the preuniversal treatment era are critical in informing this area of research.

Adults with LTNP are often defined as those who, in the absence of ART, maintain CD4^+^ cell counts above a threshold (often 500 cells/μl) without clinical disease progression over a specified duration after HIV diagnosis, and/or criteria relating to changes in CD4^+^ cell count over time [4,10]. For CWHIV, definitions of LTNP vary but often involve surviving to a specified age (often 8 years [11–15]) without ART, clinical (e.g. CDC stage B or C events) or immunological (e.g. CD4^+^ cell count <500 cells/μl) events.

Differences in definitions, study design and inclusion criteria can lead to differences in estimates of the prevalence of LTNP [16–18]. Cross-sectional analyses of CWHIV who have survived to a given age can estimate the prevalence of LTNP amongst these survivors [11–13] but not the probability that a child with perinatal HIV will go on to have LTNP (as children who die earlier are excluded). The latter is best estimated in longitudinal birth cohorts [17], although these exclude children who are diagnosed later in childhood, whose mothers may have been undiagnosed during pregnancy or not engaged in care, potentially leading to selection bias. Dynamic cohorts including CWHIV of any age as they enter care are an alternative approach but may be subject to survivor bias, as some children may die before HIV diagnosis. Further methodological challenges arise in the universal treatment era, as historically ART initiation has been used as a proxy for clinical progression [19–22], but this approach has become less valid in the era of universal ART irrespective of clinical status.

In this study, we pooled individual patient data on CWHIV from long-standing cohorts across 13 European countries and Thailand to estimate the probability of LTNP among children born before the universal ART era [23]. We explored the effect of different definitions and inclusion criteria on these estimates.

## Methods

### Data source

The European Pregnancy and Paediatric Infections Cohort Collaboration (EPPICC) is a network of observational cohorts of pregnant women and children living with infections, including HIV, in Europe and Thailand [24]. EPPICC includes single and multicentre cohorts of large paediatric HIV referral centres, as well as national cohorts including data on all children in HIV care in the country. Routine care data (including demographic, clinical and treatment data) are pooled using a standardized format based on the HIV Cohorts Data Exchange Protocol (HICDEP, www.hicdep.org). This analysis used data from the EPPICC 2016 paediatric data merger, with follow-up data from cohort inception to October 2016 (Supplementary Table 1, Supplementary Digital Content).

### Inclusion criteria for analysis

Children with perinatally acquired HIV infection (recorded as perinatally acquired or unknown mode of acquisition and presenting to care aged <10 years) were included if they:

1. Were born domestically (in the country of the cohort), and
2. Had at least two CD4^+^ cell count or CD4% measurements (as appropriate to the definition of LTNP, see below) with less than 15 months between measurements, and
3. Were born before 2008 for analysis of LTNP defined at age 8 (2011 for LTNP at age 5), for ascertainment of LTNP status at the appropriate age.

The main analysis included children born domestically (to avoid survivorship bias for children born abroad) and was restricted to cohorts with national coverage (Italy, the Netherlands, Spain, Switzerland, United Kingdom and Ireland). Children with a recorded AIDS diagnosis but without a diagnosis date were excluded.

### Definitions of disease progression and long-term nonprogression

Date of disease progression was the earliest of confirmed immunological progression, AIDS diagnosis or death (irrespective of immunological status). LTNP was defined as not experiencing disease progression, or ART initiation, before the age of 5 or 8 years. ART initiation was accounted for by treating it as a competing event in the statistical analysis (see below).

We explored four definitions of immunological progression, using two consecutive measurements showing: WHO advanced or severe immunosuppression for age (CD4% <30% for children aged <11 months, <25% for 12–35 months, <20% for 36–59 months, and CD4% <15% or CD4^+^ cell count <200 cells/μl for >5 years) [25], age-adjusted CD4^+^*z*-scores <−2 (see Supplementary Methods [26], Supplementary Digital Content), age-adjusted CD4^+^*z*-scores <  −3 [26], CD4^+^ cell counts < 500 cells/μl. We estimated the date on which the CD4^+^ count, CD4^+^*z*-score or CD4% fell below the threshold for progression using linear interpolation (Supplementary Methods, Supplementary Digital Content). Children who met an immunological threshold on a single measurement and started ART within 120 days were considered to have progressed on the (interpolated) date of immunological progression.

Thus eight definitions of LTNP (four immunological criteria at two different ages) were explored.

### Statistical analysis

We used competing risk approaches (cause-specific hazards) to estimate the cumulative incidence function (CIF) for disease progression in the absence of ART according to age, with ART initiation as a competing event [27]. These models estimate the probabilities (CIFs) of two outcomes: disease progression without prior ART initiation and ART initiation without prior evidence of disease progression. Children with neither of these outcomes by the age of 8 (or 5) years were considered to have LTNP (i.e. children who had not initiated ART and had no evidence of disease progression were classified as having LTNP). The probability of LTNP was estimated as 100% minus the sum of the two CIFs, with 95% confidence intervals (CIs) based on the confidence limits of the Kaplan–Meier estimate of the survival function for the composite outcome of progression or ART initiation.

Children were considered at risk of progression from birth until earliest of progression, ART initiation or last CD4^+^ measure (or the last before a gap of >15 months). Those who met an immunological threshold for the first time at their last recorded measurement were censored at the previous measurement as progression required two consecutive measures below the threshold.

In the main analysis (children born domestically in national cohorts), we estimated the probability of LTNP for each of the eight definitions. We then described characteristics of children with LTNP, focusing on the definition of LTNP at age 8 with disease progression based on WHO immunosuppression. Amongst those with LTNP who had at least three viral load measurements over at least 12-month period, we estimated the percentage who had at least one episode of ‘elite control’ before the age threshold (defined as ≥3 consecutive viral load<50 copies/ml, or below the lower limit of detection, over ≥12 months [28]).

Characteristics of children who did and did not meet the definition of LTNP were compared in an exploratory analysis using *χ*^2^ tests and Wilcoxon rank sum tests.

We also described subsequent outcomes amongst children with LTNP by summarizing their immune status, viral load and height-for-age *z*-scores (HAZ, calculated with WHO Child Growth Charts [29]) at yearly intervals following the age threshold, using the measurement closest to the relevant birthday (within 90 days). We summarized CD4^+^ cell count, viral load and HAZ trajectories for children with no recorded progression or ART initiation by age 18 years.

Analyses are stratified by year of birth [pre-1997, 1997–2003, 2004–2007, 2008–2011 (the latter for age 5 years definitions only)]. This reflects increasing inclusivity of paediatric treatment guidelines/practice (e.g. initiating treatment in infants aged less than 12 months if CD4% was less than 20% in 2003, expanding to immediate treatment of all infants irrespective of immune status following publication of the CHER trial in 2008 [30], and universal treatment of all children irrespective of age and immune/clinical status in 2013 [23]).

In additional analyses, we used alternative inclusion criteria and estimated the probability of LTNP at age 8 using WHO immunosuppression categories. First, we restricted analysis to children born domestically in national cohorts and with at least one CD4 measurement before 12 months of age (as a proxy for being in HIV care during infancy, when risk of disease progression is highest). Second, we widened the inclusion criteria to all children born domestically from all cohorts (national and nonnational), irrespective of age at first entry.

Analyses were carried out in Stata (v18.0, StataCorp LLC, College Station, Texas, USA).

### Ethics approval

EPPICC has ethics approval from the UCL Research Ethics Committee (reference 17493/001). Each collaborating cohort obtained local ethical approval/exemptions.

## Results

The EPPICC paediatric 2016 dataset included data on 9666 children from 18 cohorts (Supplementary Table 1, Supplementary Digital Content).

### Analyses including all children born domestically and enrolled in national cohorts

**Fig. 1:**
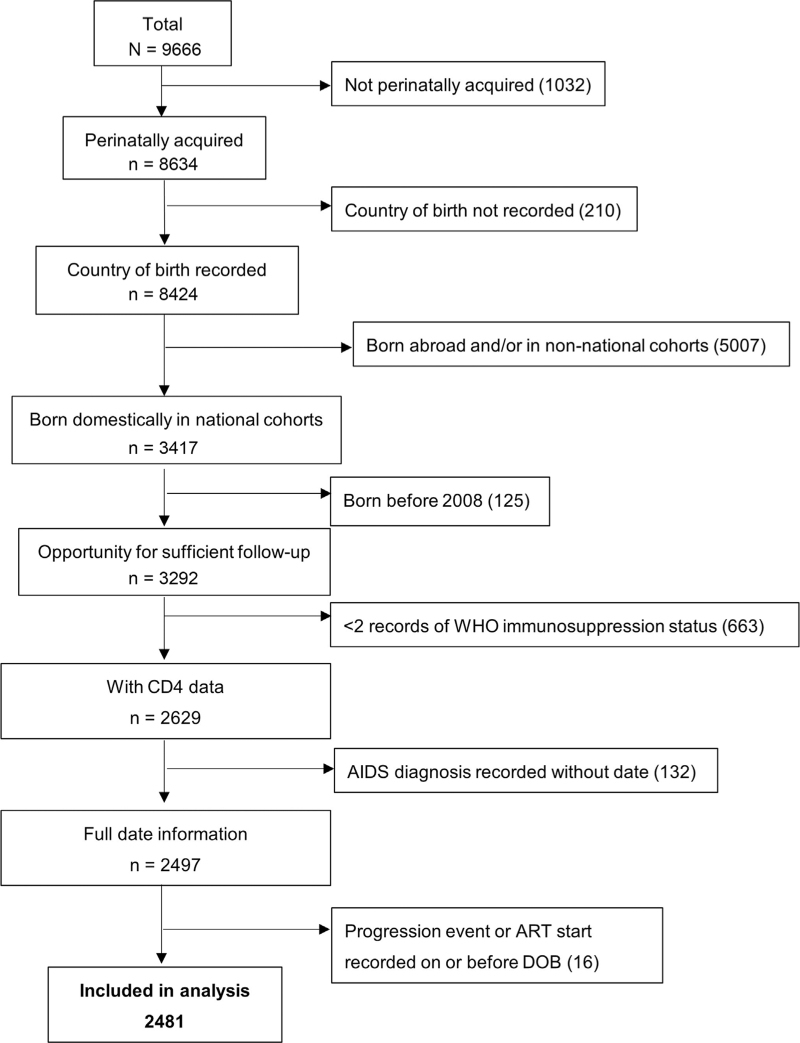
Participant flow chart (main analysis).

For the main analysis, which included children born domestically and enrolled in national cohorts, 2455–2558 children from six cohorts were included, depending on the age cut-off and immunological threshold in the LTNP definition (Table 1). For the analysis defining LTNP at age 8 using WHO categories, participant flow is shown in Fig. 1. Of 2481 children included in this analysis, 1306 (53%) were girls and most (1538, 62%) were born before 1997 (Table 2). A third (30%) were in the UK/Ireland and the remainder elsewhere in Europe. Almost all children (98%) had perinatally acquired HIV; for those with unknown mode of acquisition, median age at HIV diagnosis was 4.4 years [IQR 1.9–7.0]. Participants were first seen in care at a median age of 0.4 years [IQR 0.0–2.2], with median follow-up duration 6.4 years [IQR 2.0–13.2]. Characteristics of children included in analyses using the same inclusion criteria, but different definitions of LTNP, were similar.

**Table 1:** Number of children with HIV included in analyses using different definitions of long-term nonprogression and including children born domestically and enrolled in national cohorts.

	Number of children included in analysis	
Definition of LTNP^∗^	LTNP at age 5 years	LTNP at age 8 years
WHO immunosuppression categories	2558	2481
CD4^+^*z*-score <−2	2550	2456
CD4^+^*z*-score <−3	2550	2456
CD4^+^ count <500 cells/μl	2549	2455

∗ Additional analyses using this definition included 1144 children born domestically and enrolled in national cohorts with their first CD4^+^ measurement before age 12 months, and 4542 children born domestically in national and nonnational cohorts.

**Table 2:** Characteristics of children included in the main analysis by long-term nonprogression status at age 8, using the definition of immune progression based on WHO immunosuppression for age status.

Characteristic		Total (*n* = 2481)	LTNP (*n* = 290)	Non-LTNP (*n* = 2191)	*P* value^∗^
		*N* (%), median [IQR]				
Sex	Male	1174 (47.3)	119 (41.0)	1055 (48.2)	0.02
	Female	1306 (52.7)	171 (59.0)	1135 (51.8)	
Year of birth	Before 1997	1538 (62.0)	207 (71.4)	1331 (60.7)	0.001
	1997–2003	700 (28.2)	68 (23.4)	632 (28.8)	
	2004 or later	243 (9.8)	15 (5.2)	228 (10.4)	
Region	UK/Ireland	753 (30.4)	144 (49.7)	609 (27.8)	<0.001
	Rest of Europe	1728 (69.6)	146 (50.3)	1582 (72.2)	
Mode of HIV acquisition	Perinatal	2442 (98.4)	277 (95.5)	2165 (98.8)	<0.001
	Unknown	39 (1.6)	13 (4.5)	26 (1.2)	
Year first seen in cohort		1996 [1992– 2001]	2000 [1993–2005]	1996 [1992–2001]	<0.001
Age first seen in cohort (years)		0.4 [0.0–2.2]	7.0 [1.8–10.0]	0.3 [0.0–1.5]	<0.001
Age at first CD4^+^ measurement (years)		1.3 [0.3–4.1]	9.2 [6.0–11.3]	0.9 [0.3–3.0]	<0.001
Number of CD4^+^ measurements during follow-up		12 [4–39]	21 [8–34]	10 [3–41]	<0.001
Ever on ART during follow-up		1922 (77.5)	216 (74.5)	1706 (77.9)	0.20
Age at ART initiation (years)		1.8 [0.5–5.0]	11.3 [9.8–13.4]	1.3 [0.4–3.6]	<0.001
Year of ART initiation	Before 1997	810 (35.6)	26 (11.2)	784 (38.3)	<0.001
	1997–2003	963 (42.3)	80 (34.3)	883 (43.2)	
	2004–2007	311 (13.7)	48 (20.6)	263 (12.9)	
	2008 or later	194 (8.5)	79 (33.9)	115 (5.6)	
Duration of follow-up (years)		6.4 [2.0–13.2]	8.5 [4.4–13.2]	5.8 [1.8–13.2]	0.0001

Children were included if they were born domestically and enrolled in cohorts with national coverage.

∗ *P* values were obtained from χ^2^ or Wilcoxon rank sum tests for categorical and continuous data, respectively. ART, antiretroviral therapy; LTNP, long-term nonprogression; WHO, World Health Organization.

**Fig. 2:**
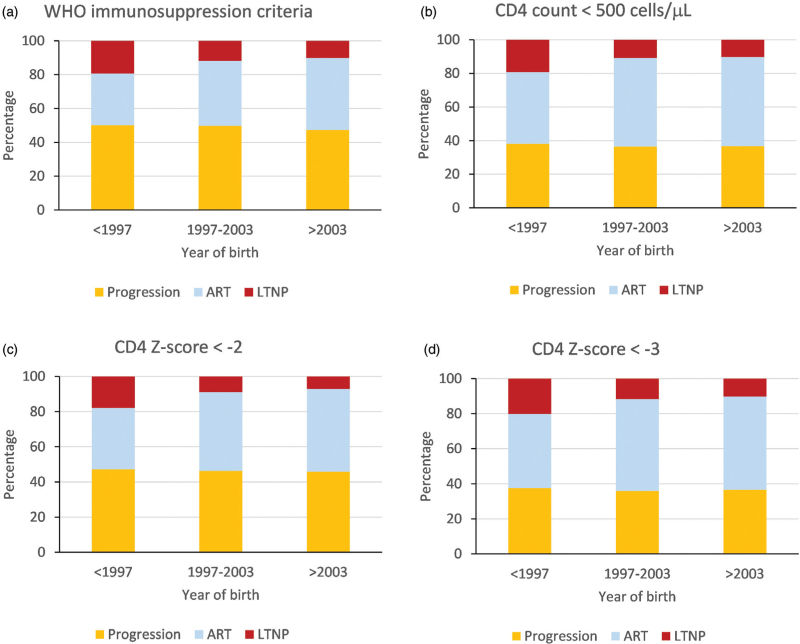
Probability of different outcomes (progression, antiretroviral therapy initiation or long-term nonprogression) in children with HIV by birth year category, with long-term nonprogression defined at age 8 using different immunological criteria (main analysis).

The probability of meeting the definition of LTNP, based on WHO categories at age 8, declined with later year of birth, from 19% (95% CI 17–22%) amongst those born before 1997 to 10% (95% CI 6–15%) amongst those born in 2004–2007 (Fig. 2a). This decline was largely due to higher probability of ART initiation (consistent with changes in treatment guidelines), and a slight concomitant decrease in the probability of progression, over time. Estimates of the probability of LTNP at age 8, and trends over birth year category, were similar across the immunological progression definitions used (Fig. 2b–d). The probability of LTNP defined at age 5 was approximately twice as high (>30%), compared to definitions at age 8 for the corresponding birth years, and much lower (<5%) among children born after 2007 due to a markedly higher probability of initiating ART (Supplementary Figure 1, Supplementary Digital Content).

The main analysis included children born domestically and enrolled in cohorts with national coverage. ART, antiretroviral therapy; CWHIV, children with HIV; LTNP, long-term nonprogression.

### Characteristics and outcomes of children with long-term nonprogression

Children meeting the definition of LTNP at age 8 years (based on WHO immunosuppression categories) were more likely to be female, born in earlier years, in the UK/Ireland cohort, older at first presentation to care, with unknown mode of HIV acquisition, compared to those not meeting this definition (Table 2, all *P* ≤ 0.02). Results were broadly similar when using other definitions of LTNP, although the sex difference was not apparent when using an age threshold of 5 years. Of 290 children who met the definition of LTNP, 40 (14%) had sufficient virological data before ART initiation to be assessed for elite control status, of whom two (5%) met the criteria for elite control at any time before age 8.

The probability of remaining progression-free and ART-naive declined after age 8, as the probabilities of progression and ART initiation increased (Supplementary Figure 2, Supplementary Digital Content). By age 10 and 12 years, 188 (65%) and 103 (36%), respectively, of the 290 children with LTNP had neither progressed nor initiated ART.

Amongst children with LTNP at age 8, the percentage with advanced or severe immunosuppression generally increased with age beyond age 8, while median viral load amongst those not virologically suppressed declined (Supplementary Figure 3, Supplementary Digital Content). Median HAZ was close to zero at all ages (Supplementary Figure 3, Supplementary Digital Content). Of 69 children with LTNP at age 8 and still in follow-up at age 18, seven (10%) had neither progressed (based on WHO immunosuppression categories) nor started ART by that point (their CD4, HAZ and viral load trajectories are shown in Supplementary Figures 4–6, Supplementary Digital Content). None of these seven met the criteria for elite control before the age of 8, although two had subsequent episodes of elite control.

### Analyses using other inclusion criteria

Additional analyses were (a) restricted to 1144 children born domestically in national cohorts and in HIV care from infancy, and (b) extended to include 4542 children born domestically in all cohorts in EPPICC, including nonnational cohorts and irrespective of age at entry to HIV care (Supplementary Table 2, Supplementary Digital Content). Their characteristics were broadly similar to the main analysis, although median [IQR] age at first presentation was younger in (a) at 0.0 years [0.0–0.3] and older in (b) at 1.4 years [0.2–4.2], compared to the main analysis.

Estimates of LTNP at age 8 years were lowest in analysis (a) at 2–4% (versus 10–19% in the main analysis), without a clear trend over time (Table 3, Supplementary Figure 7 Supplementary Digital Content). In analysis (b), the probability of LTNP was higher for earlier calendar years of birth [e.g. 31% (95% CI 29–33%) for those born before 1997] but was similar to the main analysis for those born in 2004 or later.

**Table 3:** Estimates of the probability of long-term nonprogression using different inclusion criteria, with 95% confidence intervals.

	Children born domestically in national cohorts (main analysis)		Children born domestically in national cohorts with first CD4^+^ before 12 months		All children born domestically (all cohorts)	
Year of birth	*N*	Probability of LTNP (%)	*N*	Probability of LTNP (%)	*N*	Probability of LTNP (%)
<1997	1538	19 (17–22)	628	4 (2–7)	1897	31 (29–33)
1997–2003	700	12 (10–15)	374	2 (1–4)	1586	17 (15–19)
2004–2007	243	10 (6–15)	142	3 (1–7)	1059	10 (8–12)
Total	2481		1144		4542	

LTNP was defined at age 8 using WHO immunosuppression categories. LTNP, long-term nonprogression.

## Discussion

In this large multinational cohort of CWHIV, an estimated 10% of children with perinatally acquired HIV born in 2004–2007 had LTNP at age 8. This estimate varied little with the different immunological criteria used but was higher with a lower age threshold, and depended on inclusion criteria. Given current recommendations for universal ART, large historic datasets such as this provide a unique opportunity to explore different definitions of LTNP and how trends over time reflect changes in guidelines on ART initiation. In our cohort, declines in the probability of LTNP were driven mainly by increases in the probability of ART initiation (reflecting changes in guidelines) while risk of disease progression remained stable over time.

Cross-sectional studies of LTNP in CWHIV [11–13,31] are difficult to compare to our longitudinal analyses. A French national birth cohort study of infants reported a 2.4% probability of LTNP at age 10 (having no more than one CD4% <25%, no CDC category B or C events, and never receiving ART other than zidovudine monotherapy) [17], whilst a similar approach in an Italian cohort estimated a 3.5% probability of remaining asymptomatic at age 8 [15]. These studies are most similar to our analysis restricted to children born domestically in national cohorts who were in HIV care from infancy, which produced a similar estimate of 2–4% probability of LTNP at age 8. Such approaches include all children with HIV diagnosed at birth or in infancy but exclude children diagnosed later, potentially underestimating the probability of LTNP as those without disease progression may be more likely to remain undiagnosed. The reasons for HIV testing were not captured in our study, but in many settings, there were efforts to test clinically well children following HIV diagnosis of a family member [32].

Estimates from our main analysis may be more representative, as they include all children in national cohorts, irrespective of age at first presentation. However, there is a small risk that children diagnosed at older ages may have incomplete clinical histories (e.g. missing details of potential disease progression events), leading to overestimation of LTNP. The inclusion of only national cohorts in the main analysis benefits from increased data completeness and ascertainment of progression, as all visits in all clinics are captured (in particular for children in shared care in local and referral centres). Our additional analyses including data from all (national and nonnational) cohorts, therefore, has an increased risk of overestimating the probability of LTNP because of higher risk of missing data. Thus, most methods of estimating the probability of LTNP are imperfect, and the true value probably lies within the range of estimates from our main and additional analyses. Additionally, there is no consensus on the definition of LTNP in adults or children, so we explored several definitions comparable to those used previously [4,10].

Most children with LTNP at age 8 years went on to experience some degree of disease progression and/or initiate ART. In our main analysis, only seven children with LTNP remained in follow-up at age 18 years without a record of disease progression or ART initiation. Although this number is small (and data before age 8 were limited for these children), it demonstrates the possibility of long-term survival with HIV without symptoms or major immunological impairment, and reinforces the importance of index case linked testing of children at risk of having perinatally acquired HIV irrespective of age and clinical status [33].

Only two children with LTNP had documented sustained undetectable viral load (elite control) without ART before age 8. Of those who remained progression-free and ART-naive at age 18, a further two had episodes of elite control after age 8. Other data also show that the vast majority of children [17,34] and adults [35] have detectable viral load despite immunological nonprogression. Previous studies have also shown that most children [31] and adults [36,37] with LTNP ultimately do experience disease progression and/or initiate ART.

Whilst it was beyond the scope of this study to identify causes or predictors of LTNP, several characteristics differed between those with and without LTNP in exploratory univariate analysis. Females were over-represented amongst those with LTNP, which has previously been reported in studies of elite control and slow progression in children [28,38]. Children from the UK/Ireland also appeared more likely to have LTNP compared to those in other cohorts, which may reflect differences in clinical practice regarding ART initiation [39] or rates of comorbidities. Children with LTNP were less likely to be born in later years, reflecting lower probability of initiating ART in earlier calendar periods. This trend may also reflect the impact of improved coverage and effectiveness of interventions to prevent vertical transmission, as infants who acquire HIV vertically despite such interventions might be least likely to develop LTNP.

Strengths of the study include use of data from a large, diverse cohort collaboration with extensive longitudinal follow-up primarily in the preuniversal ART era, with regular CD4^+^ and viral load assessments offering an opportunity to assess the probability of LTNP in the absence of ART. Except for CD4^+^ cell count less than 500 cells/μl, the definitions of immunosuppression used account for age, and we used statistical methods which treat ART initiation as a competing risk.

Limitations include the relatively high median age at presentation to HIV care amongst those meeting the definition of LTNP. Some children may have experienced progression events before presentation, which were not recorded in our dataset, leading to potential overestimation of LTNP, although this is less likely in national cohorts, and late diagnosis without disease progression is consistent with LTNP. There is a small possibility that some children may have died before they were diagnosed with HIV, or before having two CD4^+^ measurements, especially in earlier years, again leading to overestimation of LTNP.

Children with LTNP who did not have clinical progression may still have sub-optimal health outcomes, although most maintained good immune status (no or mild immunosuppression) and average growth, and many clinically important issues such as severe infections, severe wasting or stunting would likely have been captured as an AIDS diagnosis.

In the current era of universal ART prior to disease progression, it is not possible to ethically assess the probability of LTNP in the absence of ART. Our dataset is largely from a preuniversal ART period and as guidelines changed toward earlier treatment initiation, the changing probability of ART initiation was addressed using competing risks analyses. Results based on these methods depend on patterns of ART use as well as progression, which are setting-specific and may not be generalizable to other regions with different patterns of ART use/access or areas with higher prevalence of childhood comorbidities (e.g. malnutrition or tuberculosis). We have not assessed the effect of demographic (e.g. region or ethnicity) or clinical factors on the probability of LTNP, and so the generalizability of our estimates to other populations is not clear. Lastly, we restricted analysis to children born domestically and excluded children who were born abroad to avoid survivorship bias.

In conclusion, this is one of the largest studies to date on LTNP in children with HIV. Our findings highlight the critical importance of considering the inclusion criteria and definitions used when analysing and interpreting studies of LTNP, as well as methodological issues arising. Our findings and proposed definitions may inform estimates of nonprogression in untreated children as well as efforts to identify children for inclusion in future studies of posttreatment control and HIV cure.

## Acknowledgements

We thank all the patients for their participation in these cohorts, and the staff members who cared for them.

The European Pregnancy and Paediatric Infections Cohort Collaboration (EPPICC):

**EPPICC/PENTA Co-ordinating Team**: Elizabeth Chappell, Siobhan Crichton, Intira Jeannie Collins, Giorgia Dalla Valle, Charlotte Duff, Carlo Giaquinto, Charlotte Jackson, Ali Judd, Laura Mangiarini, Claire Thorne.

**Collaborating cohorts:**Belgium: Hopital St Pierre Cohort, Brussels: Tessa Goetghebuer, MD, PhD; Marc Hainaut, MD PhD; Wivine Tremerie, Research nurse; Marc Delforge, data manager.

Greece: Greek cohort: Vana Spoulou.

Italy: Italian Register for HIV infection in Children. Coordinators: Maurizio de Martino, Luisa Galli (Florence), Pier Angelo Tovo, Clara Gabiano (Turin). Participants: Ines Carloni (Ancona), Domenico Larovere (Bari), Francesco Baldi, Angela Miniaci, Andrea Pession (Bologna), Raffaele Badolato (Brescia), Grazia Pantò (Catania), Elisa Anastasio (Catanzaro), Carlotta Montagnani, Elisabetta Venturini, Leila Bianchi (Florence), Alessandra Allodi, Antonio Di Biagio, Sara Grignolo (Genua), Vania Giacomet, Paola Marchisio, Giuseppe Banderali, Claudia Tagliabue (Milan), Monica Cellini (Modena), Eugenia Bruzzese, Pasquale Di Costanzo, Andrea Lo Vecchio (Naples), Carlo Giaquinto, Daniele Donà, Osvalda Rampon (Padua), Amelia Romano (Palermo), Icilio Dodi, Susanna Esposito (Parma), Valentina Zuccaro, Domenico Zanaboni (Pavia), Rita Consolini (Pisa), Stefania Bernardi, Orazio Genovese (Rome), Letizia Cristiano (Taranto), Antonio Mazza (Trento), Silvia Garazzino, Federica Mignone, Erika Silvestro (Turin), Vincenzo Portelli (Trapani).

Latvia: Latvian Cohort (Santa Ansone).

Netherlands: The ATHENA cohort is managed by Stichting HIV Monitoring and supported by a grant from the Dutch Ministry of Health, Welfare and Sport through the Centre for Infectious Disease Control of the National Institute for Public Health and the Environment.

CLINICAL CENTRES (PAEDIATRIC CARE)

Emma Kinderziekenhuis (Amsterdam UMC, AMC site): *HIV treating physicians:* M. van der Kuip, D. Pajkrt H.J. Scherpbier*. HIV nurse consultants:* C. de Boer, A.M. Weijsenfeld. *HIV clinical virologists/chemists:* S. Jurriaans, N.K.T. Back, H.L. Zaaijer, B. Berkhout, M.T.E. Cornelissen, C.J. Schinkel, K.C. Wolthers. Erasmus MC–Sophia, Rotterdam: *HIV treating physicians:* P.L.A. Fraaij, A.M.C. van Rossum, C.L. Vermont. *HIV nurse consultants:* L.C. van der Knaap, E. Visser. *HIV clinical virologists/chemists:* C.A.B. Boucher, M.P.G Koopmans, J.J.A van Kampen. Radboudumc, Nijmegen: *HIV treating physicians:* S.S.V. Henriet, M. K. van Aerde. *HIV nurse consultants:* R. Strik-Albers. *HIV clinical virologists/chemists:* J. Rahamat-Langendoen, F.F. Stelma. *HIV clinical pharmacology consultant:* D. Burger. Universitair Medisch Centrum Groningen/Beatrix Kinderziekenhuis, Groningen: *HIV treating physicians:* E.H. Schölvinck, A.R. Verhage. *HIV nurse consultants:* H. de Groot-de Jonge. *HIV clinical virologists/chemists:* H.G.M. Niesters, C.C. van Leer-Buter, M. Knoester. Wilhelmina Kinderziekenhuis, UMC Utrecht, Utrecht: *HIV treating physicians:* L.J. Bont, S.P.M. Geelen, Y.G.T. Loeffen, T.F.W. Wolfs. *HIV nurse consultants:* N. Nauta. *HIV clinical virologists/chemists:* R. Schuurman, L. M Hofstra, A.M.J. Wensing.

COORDINATING CENTRE

*Director:* P. Reiss. *Deputy director:* S. Zaheri. *Data analysis:* A.C. Boyd, D.O. Bezemer, A.I. van Sighem, C. Smit, F.W.M.N. Wit. *Data management and quality control:* M.M.J. Hillebregt, T.J. Woudstra. *Data monitoring:* D. Bergsma, L. van de Sande, T. Rutkens, S. van der Vliet, K. J Lelivelt, A Scheijgrond. *Data collection:* L. de Groot, M. van den Akker, Y. Bakker, A. El Berkaoui, M. Bezemer, N. Brétin, E. Djoechro, M. Groters, E. Kruijne, K. J. Lelivelt, C. Lodewijk, E. Lucas, L. Munjishvili, F. Paling, B. Peeck, C. Ree, R. Regtop, Y. Ruijs, M. Schoorl, P. Schnörr, E. Tuijn, L. Veenenberg, K. M. Visser, E. C. Witte. *Patient registration:* Y. Ruijs.

Poland: Polish paediatric cohort: Head of the team: Prof Magdalena Marczyńska, MD, PhD Members of the team: Jolanta Popielska, MD, PhD; Maria Pokorska-Śpiewak, MD, PhD; Agnieszka Ołdakowska, MD, PhD; Konrad Zawadka, MD, PhD; Magdalena Pluta MD, PhD Administration assistant: Małgorzata Doroba. Affiliation: Medical University of Warsaw, Poland, Department of Children's Infectious Diseases; Hospital of Infectious Diseases in Warsaw, Poland.

Romania: ‘Victor Babes’ Hospital Cohort, Bucharest: Dr Luminita Ene.

Russia: Federal State-owned Institution ‘Republican Clinical Infectious Diseases Hospital’ of the Ministry of Health of the Russian Federation, St Petersburg: Liubov Okhonskaia, Evgeny Voronin, Milana Miloenko, Svetlana Labutina.

Russia: The City HIV Centre, St Petersburg: Anna Samarina.

Russia: Irkutsk AIDS Centre: Anna Turkova, Yulia Plotnikova.

Spain: CoRISPE-cat, Catalonia: CoRISPE-cat receives financial support from the Instituto de Salud Carlos III through the Red Temática de Investigación Cooperativa en Sida (grant numbers RED RIS RD06/0006/0035 yRD06/0006/0021). Members: Hospital Universitari Vall d’Hebron, Barcelona [Pere Soler-Palacín, Maria Antoinette Frick and Santiago Pérez-Hoyos (statistician)], Hospital Universitari del Mar, Barcelona (Núria López), Hospital Universitari Germans Trias i Pujol, Badalona (María Méndez, Clara Carreras), Hospital Universitari JosepTrueta, Girona (Borja Guarch), Hospital Universitari Arnau de Vilanova, Lleida (Teresa Vallmanya, Laura Minguell-Domingo), Hospital Universitari Joan XXIII, Tarragona (Olga Calavia), Consorci Sanitari del Maresme, Mataró (Lourdes García), Hospital General de Granollers (Maite Coll), Corporació Sanitària Parc Taulí, Sabadell (Valentí Pineda), Hospital Universitari Sant Joan, Reus (Neus Rius), Fundació Althaia, Manresa (Núria Rovira), Hospital Son Espases, Mallorca (Joaquín Dueñas) and Hospital Sant Joan de Déu, Esplugues (Clàudia Fortuny, Anna Gamell, Antoni Noguera-Julian).

Spain: CoRISPE-S and Madrid cohort.

Received funding from: Estudio del análisis clínico-epidemiológico de la infección por el vih en niños y adolescentes, mujeres embarazadas y sus hijos a nivel nacional. Ministerio Sanidad. Proyect 202007PN0002.

Paediatrics units: María José Mellado, Luis Escosa, Milagros García Hortelano, Talía Sainz, Carlos Grasa, Paula Rodríguez (Hospital Universitario La Paz, Madrid); Pablo Rojo, Luis Prieto-Tato, Cristina Epalza, Alfredo Tagarro, Sara Domínguez, Álvaro Ballesteros (Hospital Universitario Doce de Octubre, Madrid); José Tomás Ramos, Marta Illán, Arantxa Berzosa, (Hospital Clínico San Carlos, Madrid); Sara Guillén, Beatriz Soto (Hospital Universitario de Getafe, Madrid); María Luisa Navarro, Jesús Saavedra, Mar Santos, David Aguilera, Begoña Santiago, Santiago Jimenez de Ory (Hospital Universitario Gregorio Marañón, Madrid); Amanda Bermejo (Hospital Universitario de Móstoles, Madrid); María Penín (Hospital Universitario Príncipe de Asturias de Alcalá de Henares, Madrid); Jorge Martínez (Hospital Infantil Universitario Niño Jesús, Madrid); Katie Badillo (Hospital Universitario de Torrejón, Madrid); Ana Belén Jiménez (Hospital Fundación Jiménez Díaz, Madrid); Adriana Navas (Hospital Universitario Infanta Leonor, Madrid); Eider Oñate (Hospital Universitario Donostia, Guipúzcoa); Itziar Pocheville (Hospital Universitario Cruces, Vizcaya); Elisa Garrote (Hospital Universitario Basurto, Vizcaya); Elena Colino, Olga Afonso (Hospital Insular Materno Infantil, Gran Canaria); Jorge Gómez Sirvent (Hospital Universitario Virgen de la Candelaria, Tenerife); Mónica Garzón, Vicente Román (Hospital General, Lanzarote); Raquel Angulo (Hospital de Poniente de El Ejido, Almería); Olaf Neth, Lola Falcón (Hospital Universitario Virgen del Rocío, Sevilla); Pedro Terol (Hospital Universitario Virgen de la Macarena, Sevilla); Juan Luis Santos, Álvaro Vázquez (Hospital Universitario Virgen de las Nieves, Granada); Begoña Carazo, Antonio Medina (Hospital Regional Universitario, Málaga); Francisco Lendínez, Mercedes Ibáñez (Complejo Hospitalario Torrecárdenas, Almería); Estrella Peromingo, María Isabel Sánchez (Hospital Universitario Puerta del Mar, Cádiz); Beatriz Ruiz (Hospital Universitario Reina Sofía de Córdoba); Ana Grande (Complejo Hospitalario Universitario Infanta Cristina, Badajoz); Francisco José Romero (Complejo Hospitalario, Cáceres); Carlos Pérez, Alejandra Méndez (Hospital de Cabueñes, Asturias); Laura Calle (Hospital Universitario Central de Asturias); Marta Pareja (Complejo Hospitalario Universitario, Albacete); Begoña Losada (Hospital Virgen de la Salud, Toledo); Mercedes Herranz,(Hospital Virgen del Camino, Navarra); Matilde Bustillo (Hospital Universitario Miguel Servet, Zaragoza); Pilar Collado (Hospital Clínico Universitario Lozano Blesa, Zaragoza); José Antonio Couceiro (Complejo Hospitalario Universitario, Pontevedra); Leticia Vila (Complejo Hospitalario Universitario, La Coruña); Consuelo Calviño (Hospital Universitario Lucus Augusti, Lugo); Ana Isabel Piqueras, Manuel Oltra (Hospital Universitario La Fe, Valencia); César Gavilán (Hospital Universitario de San Juan de Alicante, Alicante); Elena Montesinos (Hospital General Universitario, Valencia); Marta Dapena (Hospital General, Castellón); Beatriz Jiménez (Hospital Universitario Marqués de Valdecilla, Cantabria); Ana Gloria Andrés (Complejo Hospitalario, León); Víctor Marugán, Carlos Ochoa (Complejo Hospitalario, Zamora); Ana Isabel Menasalvas, Eloísa Cervantes, Beatriz Álvarez (Hospital Universitario Virgen de la Arrixaca, Murcia) and Paediatric HIV-BioBank integrated in the Spanish AIDS Research Network and collaborating Centers.

**Adults Units:** Cristina Díez, (Hospital Universitario Gregorio Marañón, Madrid).

Ignacio Bernardino, María Luisa Montes, Eulalia Valencia, Ana Delgado (Hospital Universitario La Paz, Madrid); Rafael Rubio, Federico Pulido, Otilia Bisbal (Hospital Universitario Doce de Octubre, Madrid); Alfonso Monereo Alonso (Hospital Universitario de Getafe, Madrid); Juan Berenguer, Cristina Díez, Teresa Aldamiz, Francisco Tejerina, Juan Carlos Bernaldo de Quirós, Belén Padilla, Raquel Carrillo, Pedro Montilla, Elena Bermúdez, Maricela Valerio (Hospital Universitario Gregorio Marañón, Madrid); Jose Sanz (Hospital Universitario Príncipe de Asturias de Alcalá de Henares, Madrid); Alejandra Gimeno (Hospital Universitario de Torrejon, Madrid); Miguel Cervero, Rafael Torres (Hospital Universitario Severo Ochoa de Leganés, Madrid); Santiago Moreno, María Jesús Perez, Santos del Campo (Hospital Universitario Ramon y Cajal, Madrid); Pablo Ryan, Jesus Troya (Hospital Universitario Infanta Leonor, Madrid); Jesus Sanz (Hospital Universitario La Princesa, Madrid); Juan Losa, Rafael Gomez (Hospital Universitario Fundacion Alcorcon, Madrid); Miguel Górgolas (Hospital Fundacion Jimenez Diaz, Madrid); Alberto Díaz, Sara de la Fuente (Hospital Universitario Puerta de Hierro de Majadahonda, Madrid); Jose Antonio Iribarren, Maria Jose Aramburu, Lourdes Martinez (Hospital Universitario Donostia, Guipuzcoa); Ane Josune Goikoetxea (Hospital Universitario Cruces, Vizcaya); Sofia Ibarra, Mireia de la Peña (Hospital Universitario Basurto, Vizcaya); Víctor Asensi (Hospital Universitario Central de Asturias); Michele Hernandez (Hospital Universitario Insular, Gran Canaria); María Remedios Alemán, Ricardo Pelazas, María del Mar Alonso, Ana María López, Dácil García, Jehovana Rodriguez (Hospital Universitario de Canarias, Tenerife); Miguel Angel Cardenes (Hospital Universitario Doctor Negrin, Gran Canaria); Manuel A. Castaño, Francisco Orihuela, Inés Pérez, Mª Isabel Mayorga (Hospital Regional Universitario, Málaga); Luis Fernando Lopez-Cortes, Cristina Roca, Silvia Llaves (Hospital Universitario Virgen del Rocio, Sevilla); Maria Jose Rios, Jesus Rodriguez, Virginia Palomo (Hospital Universitario Virgen de la Macarena, Sevilla); Juan Pasquau, Coral Garcia (Hospital Universitario Virgen de las Nieves, Granada); Jose Hernandez, Clara Martinez (Hospital Universitario Clinico San Cecilio, Granada); Antonio Rivero, Angela Camacho (Hospital Universitario Reina Sofia, Cordoba); Dolores Merino, Miguel Raffo, Laura Corpa (Hospital Universitario Juan Ramon Jimenez, Huelva); Elisa Martinez, Fernando Mateos, Jose Javier Blanch (Complejo Hospitalario Universitario, Albacete); Miguel Torralba (Hospital Universitario, Guadalajara); Piedad Arazo, Gloria Samperiz (Hospital Universitario Miguel Servet, Zaragoza); Celia Miralles, Antonio Ocampo, Guille Pousada (Hospital Alvaro Cunqueiro, Pontevedra); Alvaro Mena (Complejo Hospitalario Universitario, La Coruna); Marta Montero, Miguel Salavert, (Hospital Universitario La Fe, Valencia); Maria Jose Galindo, Natalia Pretel (Hospital Clinico Universitario, Valencia); Joaquín Portilla, Irene Portilla (Hospital General Universitario, Alicante); Felix Gutierrez, Mar Masia, Cati Robledano, Araceli Adsuar (Hospital General Universitario de Elche, Alicante); Carmen Hinojosa, Begoña Monteagudo (Hospital Clinico, Valladolid); Pablo Bachiller (Hospital General, Segovia); Jesica Abadía (Hospital Universitario Rio Hortega, Valladolid); Carlos Galera, Helena Albendin, Marian Fernandez (Hospital Universitario Virgen de la Arrixaca, Murcia); Jose Ramon Blanco (Complejo Hospitalario San Millan-San Pedro, la Rioja).

Sweden: Karolinska University Hospital, Stockholm, The Swedish InfCareHIV cohort (Lars Navér, Sandra Soeria-Atmadja, Erik Belfrage, Vendela Hagås).

Switzerland:*Members of the Swiss HIV Cohort Study (SHCS) and the Swiss Mother and Child HIV Cohort (MoCHiV) Study:* Abela I, Aebi-Popp K, Anagnostopoulos A, Battegay M, Baumann M, Bernasconi E, Braun DL, Bucher HC, Calmy A, Cavassini M, Ciuffi A, Crisinel PA, Darling K, Dollenmaier G, Duppenthaler A, Egger M, Elzi L, Fehr J, Fellay J, Francini K, Furrer H, Fux CA, Günthard HF (President of the SHCS), Hachfeld A, Haerry D (deputy of ‘Positive Council’), Hasse B, Hirsch HH, Hoffmann M, Hösli I, Huber M, Jackson-Perry D (patient representatives), Kahlert CR (Chairman of the Mother & Child Substudy), Keiser O, Klimkait T, Kohns M, Kottanattu L, Kouyos RD, Kovari H, Kusejko K (Head of Data Centre), Labhardt N, Martinez de Tejada B, Marzolini C, Metzner KJ, Müller N, Nemeth J, Nicca D, Notter J, Paioni P, Pantaleo G, Perreau M, Polli Ch, Ranieri E, Rauch A (Chairman of the Scientific Board), Salazar-Vizcaya L, Schmid P, Speck R, Stöckle M (Chairman of the Clinical and Laboratory Committee), Tarr P, Thanh Lecompte M, Trkola A, Wagner N, Wandeler G, Weisser M, Yerly S. Funding: the Swiss HIV Cohort Study is supported by the Swiss National Science Foundation (grant #201369) and by the SHCS research foundation.

Thailand: Program for HIV Prevention & Treatment (PHPT). Participating hospitals: Lamphun: Pornpun Wannarit; Phayao Provincial Hospital: Pornchai Techakunakorn; Chiangrai Prachanukroh: Rawiwan Hansudewechakul; Chiang Kham: Vanichaya Wanchaitanawong; Phan: Sookchai Theansavettrakul; Mae Sai: Sirisak Nanta; Prapokklao: Chaiwat Ngampiyaskul; Banglamung: Siriluk Phanomcheong; Chonburi: Suchat Hongsiriwon; Rayong: Warit Karnchanamayul; Bhuddasothorn Chacheongsao: Ratchanee Kwanchaipanich; Nakornping: Suparat Kanjanavanit; Somdej Prapinklao: Nareerat Kamonpakorn, Maneeratn Nantarukchaikul; Bhumibol Adulyadej: Prapaisri Layangool, Jutarat Mekmullica; Pranangklao: Paiboon Lucksanapisitkul, Sudarat Watanayothin; Buddhachinaraj: Narong Lertpienthum; Hat Yai: Boonyarat Warachit; Regional Health Promotion Center 6, Khon Kaen: Sansanee Hanpinitsak; Nong Khai: Sathit Potchalongsin; Samutsakhon: Pimpraphai Thanasiri, Sawitree Krikajornkitti; Phaholpolphayuhasena: Pornsawan Attavinijtrakarn; Kalasin: Sakulrat Srirojana; Nakhonpathom: Suthunya Bunjongpak; Samutprakarn: Achara Puangsombat; Mahasarakam: Sathaporn Na-Rajsima; Roi-et: Pornchai Ananpatharachai; Sanpatong: Noppadon Akarathum; Vachira Phuket: Weerasak Lawtongkum; Chiangdao: Prapawan Kheunjan, Thitiporn Suriyaboon, Airada Saipanya.

Data management team: Kanchana Than-in-at, Nirattiya Jaisieng, Rapeepan Suaysod, Sanuphong Chailoet, Naritsara Naratee, and Suttipong Kawilapat.

Ukraine: Paediatric HIV Cohort: Dr T. Kaleeva, Dr Y. Baryshnikova (Odessa Regional Centre for HIV/AIDS); Dr I. Raus (Kyiv City Centre for HIV/AIDS); Dr O. Glutshenko, (Mykolaiv Regional Centre for HIV/AIDS); Dr H. Sherstiuk (Dnipropetrovsk Regional Medical Center for Socially Significant Diseases); Dr I. Shkurka (Center for the Prevention of HIV infection/AIDS and hepatitis of Chernihiv Regional Hospital); Dr L. Knyshuk (Lviv Regional Phtysiopulmonology Cinical Center); Dr N. Delikhovska (Khmelnytsky Regional Center for the Prevention of HIV infection/AIDS); Dr I. Popova (Zaporizhzhia Center for the Prevention of HIV infection/AIDS); Dr T. Golubieva (Poltava Regional Center for the Prevention of HIV infection/AIDS); Dr Alla Volokha (Shupyk National Healthcare University of Ukraine); Dr Ruslan Malyuta (Perinatal Prevention of AIDS Initiative, Odessa); Dr H. Bailey, Prof Claire Thorne (UCL, London, UK). Funding acknowledgement: PENTA Foundation.

UK & Ireland: Collaborative HIV Paediatric Study (CHIPS): CHIPS was funded by the NHS (London Specialised Commissioning Group) and received additional support from Abbott, Boehringer Ingelheim, Bristol-Myers Squibb, GlaxoSmithKline, Gilead Sciences, Janssen and Roche. The MRC Clinical Trials Unit at UCL is supported by the Medical Research Council (https://www.mrc.ac.uk) programme number MC_UU_00004/03.

**CHIPS Steering Committee:** Hermione Lyall (chair), Alasdair Bamford, Karina Butler, Katja Doerholt, Conor Doherty, Caroline Foster, Ian Harrison, Julia Kenny, Nigel Klein, Gillian Letting, Paddy McMaster, Fungai Murau, Edith Nsangi, Katia Prime, Andrew Riordan, Fiona Shackley, Delane Shingadia, Sharon Storey, Gareth Tudor-Williams, Anna Turkova, Steve Welch. *MRC Clinical Trials Unit:* Intira Jeannie Collins, Claire Cook, Siobhan Crichton, Donna Dobson, Keith Fairbrother, Diana M. Gibb, Ali Judd, Marthe Le Prevost, Nadine Van Looy. *Integrated Screening Outcome Surveillance Service (ISOSS)***, UCL:** Helen Peters, Kate Francis, Claire Thorne.

**Hospitals participating in CHIPS in 2019/20:** University Hospitals Birmingham NHS Foundation Trust, Birmingham: L Thrasyvoulou, S Welch; Brighton and Sussex University Hospitals NHS Trust: K Fidler; University Hospitals Bristol NHS Foundation Trust, Bristol: J Bernatoniene, F Manyika; Calderdale and Huddersfield NHS Foundation Trust, Halifax: G Sharpe; Derby Teaching Hospitals NHS Foundation Trust: B Subramaniam; Glasgow Royal Hospital for Children, Glasgow: R Hague, V Price; Great Ormond Street Hospital for Children NHS Foundation Trust, London: J Flynn, N Klein, A Bamford, D Shingadia, K Grant; Oxford University Hospitals NHS Foundation Trust, Oxford: S Yeadon, S Segal; King's College Hospital NHS Foundation Trust, London: S Hawkins; Leeds Teaching Hospitals NHS Trust, Leeds: M Dowie; University Hospitals of Leicester NHS Trust, Leicester: S Bandi, E Percival; Luton and Dunstable Hospital NHS Foundation Trust, Luton: M Eisenhut; K Duncan; Milton Keynes General University Hospital NHS Foundation Trust, Milton Keynes: L Anguvaa, L Wren, Newcastle upon Tyne Hospitals NHS Foundation Trust, Newcastle: T Flood, A Pickering; The Pennine Acute Hospitals NHS Trust, Manchester: P McMaster C Murphy; North Middlesex University Hospital NHS Trust, London: J Daniels, Y Lees; Northampton General Hospital NHS Trust, Northampton: F Thompson; London North West Healthcare NHS Trust, Middlesex; A Williams, B Williams, S Pope; Barts Health NHS trust, London Dr S Libeschutz; Nottingham University Hospitals NHS Trust, Nottingham: L Cliffe, S Southall; Portsmouth Hospitals NHS Trust, Portsmouth: A Freeman; Raigmore Hospital, Inverness: H Freeman; Royal Belfast Hospital for Sick Children, Belfast: S Christie; Royal Berkshire NHS Foundation Trust, Reading: A Gordon; Royal Children's Hospital, Aberdeen: D Rosie Hague, L Clarke; Royal Edinburgh Hospital for Sick Children, Edinburgh: L Jones, L Brown; Royal Free NHS Foundation Trust, London: M Greenberg; Alder Hey Children's NHS Foundation Trust, Liverpool: C Benson, A Riordan; Sheffield Children's NHS Foundation Trust, Sheffield: L Ibberson, F Shackley; University Hospital Southampton NHS Foundation Trust, Southampton: S Patel, J Hancock; St George's University Hospitals NHS Foundation Trust, London: K Doerholt, K Prime, M Sharland, S Storey; Imperial College Healthcare NHS Trust, London: EGH Lyall, C Foster, P Seery, G Tudor-Williams, N Kirkhope, S Raghunanan; Guy's and St Thomas’ NHS Foundation Trust, London: Dr Julia Kenny, A Callaghan; University Hospitals of North Midlands NHS Trust, Stoke On Trent: A Bridgwood, P McMaster; University Hospital of Wales, Cardiff: J Evans, E Blake; NHS Frimley Health Foundation Trust, Slough: A Yannoulias.

Author contributions: I.J.C., R.G., S.C. and A.J. conceived the study. C.J. led the analysis and drafted the article, with substantial input from S.C., R.G., I.J.C. and A.J. A.B., P.G., N.K. and V.A.V. provided guidance on interpretation. L.M., P.P., A.R., V.S., S.A., E.C., S.L.C., L.E., L.G., C.G., T.G., C.F., S.K., M.M., M.N., L.N., N.N.-G.-H., Y.K.P., A.A.P., J.T.R., I.R., V.Y.R, A.V.S., E.H.S., N.V. and A.V. contributed to data collection in the individual cohorts within EPPICC. I.J.C. and A.J. are co-Principal Investigators of EPPICC. All authors provided intellectual input and critically reviewed the manuscript.

Data sharing statement: the datasets analysed during the current study are not publicly available due to the highly sensitive nature of the data (inclusion of HIV status) but are available on reasonable request to mrcctu.ctuenquiries@ucl.ac.uk.

### Conflicts of interest

EPPICC is a collaboration between University College London and Fondazione Penta ETS (http://penta-id.org). Some EPPICC activities receive industry funding, including ViiV Healthcare and Gilead Sciences during the time this work was carried out. The MRC Clinical Trials Unit at UCL is supported by the Medical Research Council (programme number: MC_UU_00004/03).

V.A.V. declares that he is currently a Gilead Sciences Inc employee and holds company's stock, but activities in this study relate to his previous position at the University of Oxford. All other authors report no conflicts of interest.

## Supplementary Material

Supplemental Digital Content

## Supplementary Material

Supplemental Digital Content

## Supplementary Material

Supplemental Digital Content

## Supplementary Material

Supplemental Digital Content

## Supplementary Material

Supplemental Digital Content

## Supplementary Material

Supplemental Digital Content

## Supplementary Material

Supplemental Digital Content

## Supplementary Material

Supplemental Digital Content

## Supplementary Material

Supplemental Digital Content

## Supplementary Material

Supplemental Digital Content
