# Supplementary material for: Long-term non-progression in children with HIV: estimates from international cohort data
Source: AIDS. 2025 Feb 4;39(6):746–59. doi: 10.1097/QAD.0000000000004136 (PMC11970603; doi:10.1097/QAD.0000000000004136)
Supplement: Supplemental Digital Content [file aids-39-746-s001.docx]

**Long-term non-progression in children living with HIV: estimates from international cohort data**

***Supplementary Digital Content: Methods***

*Further information on CD4 z-scores*

CD4 z-scores were calculated from CD4 counts based on the approach of Wade and Ades and taking HIV exposed uninfected children as the reference population (1). The z-score expresses the CD4 count as the number of standard deviations by which it differs from the expected value for a child of a given age; a z-score of 0 corresponds to the expected CD4 count, with positive and negative values indicating values which are higher and lower than expected, respectively. If a CD4 count was recorded as zero, the CD4 z-score was assigned as -12 (to reflect severe immunosuppression). If a participant had more than one measurement recorded on the same date, the lowest CD4 count (or more severely suppressed immunosuppression category) was used.

*Linear interpolation of dates of immunological progression*

To estimate the date on which each patient’s CD4 count or CD4 z-score fell below the relevant threshold, we used linear interpolation between the date of the last measurement above the threshold and the date of the first of two (or more) below the threshold.

For the analyses based on WHO immunosuppression categories, interpolation accounted for the change in units with age (i.e. for children aged <5 years, the categorisation is based on CD4%, whereas for older children it is based on CD4 counts or CD4% if severely immunosuppressed, see Supplementary Table 1). This used the following steps, summarised in the figure below:

1. Calculate patient age at date A (date of the last CD4 count or CD4% above the threshold) and date B (date of the first of two or more below the threshold)
2. If the patient *does not* change age group (as defined for the WHO immunosuppression categories) between dates A and B, use linear interpolation to estimate the date at which the CD4 count or CD4% (as appropriate for the age group) crossed the threshold for advanced or severe immunosuppression
3. If the patient *does* change age group between dates A and B, interpolate the CD4 count or CD4% at the upper limit of the age group which the patient belongs to on date A (e.g. their 3^rd^ birthday). If this is under the threshold for advanced or severe immunosuppression, estimate the date on which the threshold was crossed by interpolating between date A and this birthday.
4. If the interpolated value on this birthday is not under the threshold, interpolate again between that birthday and date B

***References***

1. Wade AM, Ades AE. Age-related reference ranges: significance tests for models and confidence intervals for centiles. Statistics in medicine. 1994;13(22):2359-67.
