# Supplementary material for: Long-term non-progression in children with HIV: estimates from international cohort data
Source: AIDS. 2025 Feb 4;39(6):746–59. doi: 10.1097/QAD.0000000000004136 (PMC11970603; doi:10.1097/QAD.0000000000004136)
Supplement: Supplemental Digital Content [file aids-39-746-s002.docx]

**Long-term non-progression in children living with HIV: estimates from international cohort data**

*Supplementary Table 1: Summary of characteristics of cohorts contributing to the EPPICC Paediatric 2016 data merger.*

| **Cohort name** | **Country** | **Children ever in follow-up** | **Latest reported visit** |
| --- | --- | --- | --- |
| *National cohorts* |  |  |  |
| ATHENA | The Netherlands | 371 | 2016 |
| Collaborative HIV Paediatric Study (CHIPS) | UK/Ireland | 2070 | 2016 |
| CORISPE-CAT* | Spain | 234 | 2015 |
| CORISPES* | Spain | 778 | 2015 |
| Italian cohort | Italy | 1839 | 2015 |
| MOCHIV | Switzerland | 276 | 2016 |
| *Sub-national cohorts* |  |  |  |
| Greek cohort | Greece | 21 | 2016 |
| Latvian cohort | Latvia | 5 | 2013 |
| PHPT | Thailand | 877 | 2014 |
| Polish cohort | Poland | 85 | 2016 |
| Porto cohort | Portugal | 45 | 2016 |
| Victor Babes Hospital Bucharest cohort | Romania | 491 | 2015 |
| Irkutsk | Russia | 674 | 2016 |
| Republican Hospital | Russia | 174 | 2014 |
| St Petersburg City Hospital | Russia | 80 | 2016 |
| St Pierre Hospital cohort | Belgium | 164 | 2015 |
| Swedish cohort | Sweden | 122 | 2016 |
| Ukrainian cohort | Ukraine | 1360 | 2016 |
|  |  |  |  |
| **TOTAL** |  | **9666** | **2016** |

*CORISPE-CAT and CORISPE-S together provide national coverage for Spain.
