# Supplementary material for: Long-term non-progression in children with HIV: estimates from international cohort data
Source: AIDS. 2025 Feb 4;39(6):746–59. doi: 10.1097/QAD.0000000000004136 (PMC11970603; doi:10.1097/QAD.0000000000004136)
Supplement: Supplemental Digital Content [file aids-39-746-s003.docx]

**Long-term non-progression in children living with HIV: estimates from international cohort data**

*Supplementary Table 2: Characteristics of children included in the analyses using alternative inclusion criteria.*

| **Characteristic** | | **Children born domestically in national cohorts with first CD4 before 12 months (n = 1144)** | **All children born domestically (n = 4542)** |
| --- | --- | --- | --- |
| Sex | Male | 524 (45.8) | 2154 (47.5) |
|  | Female | 620 (54.2) | 2385 (52.5) |
| Year of birth | Before 1997 | 628 (54.9) | 1897 (41.8) |
|  | 1997-2003 | 374 (32.7) | 1586 (34.9) |
|  | 2004 or later | 142 (12.4) | 1059 (23.3) |
| Region | UK / Ireland | 279 (24.4) | 753 (16.6) |
|  | Thailand | 0 (0.0) | 678 (14.9) |
|  | Russia / Ukraine | 0 (0.0) | 1249 (27.5) |
|  | Rest of Europe | 865 (75.6) | 1862 (41.0) |
| Mode of HIV acquisition | Perinatal | 1138 (99.5) | 4465 (98.3) |
|  | Unknown | 6 (0.5) | 77 (1.7) |
| Year first seen in cohort |  | 1996 [1992 - 2001] | 2002 [1995 - 2006] |
| Age first seen in cohort (years) |  | 0.0 [0.0 - 0.3] | 1.4 [0.2 - 4.2] |
| Age at first CD4 count (years) |  | 0.3 [0.1 - 0.5] | 2.9 [0.6 - 6.5] |
| Number of CD4 counts |  | 10 [3 - 44] | 11 [4 - 25] |
| Ever on ART |  | 874 (76.4) | 3639 (80.1) |
| Age at ART initiation (years) |  | 0.5 [0.3 - 1.0] | 2.9 [0.8 - 6.8] |
| Year of ART initiation | Before 1997 | 420 (39.5) | 821 (19.9) |
|  | 1997-2003 | 463 (43.6) | 1398 (33.8) |
|  | 2004-2007 | 145 (13.6) | 991 (24.0) |
|  | 2008 or later | 35 (3.3) | 921 (22.3) |
| Duration of follow-up (years) |  | 4.5 [1.4 - 13.7] | 6.6 [2.6 - 11.0] |
