# Supplementary material for: Long-term non-progression in children with HIV: estimates from international cohort data
Source: AIDS. 2025 Feb 4;39(6):746–59. doi: 10.1097/QAD.0000000000004136 (PMC11970603; doi:10.1097/QAD.0000000000004136)
Supplement: Supplemental Digital Content [file aids-39-746-s006.docx]

**Long-term non-progression in children living with HIV: estimates from international cohort data**

*Supplementary Figure 3: Distribution of WHO immunosuppression status, log VL and HAZ by age for children meeting the definition of LTNP (based on WHO immunosuppression categories) at age 8 years (main analysis).*

**

* The main analysis included children born domestically and enrolled in cohorts with national coverage.

Numbers contributing data each year are shown; black horizontal lines show the median. X axis values on the scatter plots are jittered to better separate the markers.

WHO, World Health Organization; VL, viral load; HAZ, height for age z-score; LTNP, long-term non-progression.
