# Supplementary material for: Long-term non-progression in children with HIV: estimates from international cohort data
Source: AIDS. 2025 Feb 4;39(6):746–59. doi: 10.1097/QAD.0000000000004136 (PMC11970603; doi:10.1097/QAD.0000000000004136)
Supplement: Supplemental Digital Content [file aids-39-746-s008.docx]

**Long-term non-progression in children living with HIV: estimates from international cohort data**

*Supplementary Figure 5: Viral load trajectories for children meeting the definition of LTNP at age 8 years and having not progressed or started ART by age 18.*
