# Supplementary material for: Long-term non-progression in children with HIV: estimates from international cohort data
Source: AIDS. 2025 Feb 4;39(6):746–59. doi: 10.1097/QAD.0000000000004136 (PMC11970603; doi:10.1097/QAD.0000000000004136)
Supplement: Supplemental Digital Content [file aids-39-746-s010.docx]

**Long-term non-progression in children living with HIV: estimates from international cohort data**

*Supplementary Figure 7:* *Percentage of CLWHIV with different outcomes (progression, ART initiation or LTNP) by birth year category, with LTNP defined at age 8 using WHO immunosuppression categories. Each panel shows results based on a different set of inclusion criteria as shown in the panel titles.*
